# 

**Published:** 1888-05

**Authors:** 


					﻿L/C AY, 1888.
OLD SERIES
Vol. XXV
NEW SERIES
Vol. V.-No. 3.\j
<5>®L,^£3
& 1855 0
Inta
■EDim*®SL
(JOURNAL
Ifeet	5

J’SWbx
W.F.WESTMORELAND.M Dj
H.V.M.MILLER.M.D.LL.D.
-ffliiBLlSHED BT JAMES P.HARRISON&CQS
fi; _____________Atlanta , ga. Sa
SUBSCRIPTION t S2.SO A YEAR.
				

